# Skeletal muscle adiponectin induction depends on diet, muscle type/activity, and exercise modality in C57BL/6 mice

**DOI:** 10.14814/phy2.13848

**Published:** 2018-10-18

**Authors:** Sergio F. Martinez‐Huenchullan, Babu R. Maharjan, Paul F. Williams, Charmaine S. Tam, Susan V. Mclennan, Stephen M. Twigg

**Affiliations:** ^1^ Greg Brown Diabetes & Endocrinology Laboratory Central Clinical School Faculty of Medicine and Health University of Sydney Sydney Australia; ^2^ School of Physical Therapy Faculty of Medicine Universidad Austral de Chile Valdivia Chile; ^3^ Department of Biochemistry School of Medicine Patan Academy of Health Sciences Lalitpur Nepal; ^4^ New South Wales Pathology Newcastle Australia; ^5^ Department of Endocrinology Royal Prince Alfred Hospital Sydney Australia; ^6^ Northern Clinical School and Centre for Translational Data Science University of Sydney Sydney Australia

**Keywords:** Adiponectin, skeletal muscle, high‐fat diet, exercise

## Abstract

Changes in skeletal muscle adiponectin induction have been described in obesity and exercise. However, whether changes are consistent across muscle types and with different exercise modalities, remain unclear. This study compared the effects of diet and two isocaloric training programs on adiponectin induction and its regulators in three muscles: *quadriceps* (exercising/glycolytic‐oxidative), *gastrocnemius* (exercising/glycolytic), and *masseter* (nonexercising/glycolytic). Ten‐week‐old male C57BL/6 mice were fed a high‐fat diet (HFD) (45% fat) or standard CHOW diet (12% fat) ad libitum and underwent one of two training regimes: (1) constant‐moderate training (END), or (2) high intensity interval training (HIIT) for 10 weeks (3 × 40 min sessions/week). Chow and HFD‐fed untrained mice were used as control. Compared with Chow, HFD induced an increase in protein levels of low‐molecular weight (LMW) adiponectin in *gastrocnemius* and *masseter* (~2‐fold; *P* < 0.05), and a decrease of high‐molecular weight adiponectin (HMW‐most bioactive form) in *quadriceps* (~0.5‐fold; *P* < 0.05). Only END prevented these changes (*P* < 0.05). HFD induced a decrease of adiponectin receptor 1 (AdipoR1) protein in exercising muscles of untrained mice (~0.5‐0.8‐fold; *P* < 0.05); notably, END also decreased AdipoR1 protein levels in lean and HFD mice. This type of training also normalized HFD‐driven mRNA changes found in some adiponectin downstream factors (sirtuin 1, Pgc‐1a, and Ucp2) in the three muscles tested. Our results indicate that diet, muscle type/activity, and exercise modality influences muscle adiponectin profile, and some of its mediators. These parameters should be taken into consideration when investigating this endocrine response of the skeletal muscle, particularly in the context of obesity and metabolic disorders.

## Introduction

Obesity is associated with skeletal muscle dysfunction (Tomlinson et al. 2014). The underlying mechanisms of this process remain poorly understood, however, it may be related to the endocrine function of skeletal muscle (Pedersen 2013). Obesity alters the ability of skeletal muscle to produce adiponectin (Yang et al. 2006; Liu et al. 2009) which could induce metabolic dysfunctions. Adiponectin has anti‐inflammatory, anti‐oxidative, and anti‐apoptotic features (Jortay et al. 2010, 2012). Given that exercise is one of the life‐style modifications proposed to counteract these pathological disturbances, our group investigated the effect of different exercise programs on *quadriceps* muscle adiponectin profiles in high‐fat fed mice. We found that 10 weeks of constant‐moderate intensity training resulted in better metabolic systemic effects which were associated with a greater increase in the most bioactive muscle adiponectin isoform (high‐molecular weight (HMW)) compared with high‐intensity interval training (Martinez‐Huenchullan et al. 2018). However, as adiponectin induction in skeletal muscles may be dependent on fiber type (Krause et al. 2008), and that the muscle group analyzed (*quadriceps)* was composed of a mixture of glycolytic and oxidative fibers (Jacobs et al. 2013), the characteristics of adiponectin induction derived from exercise between muscles with different metabolic profiles, requires exploration. Moreover, to elucidate if this exercise‐related response is localized in muscle and/or is systemic, comparisons between exercising and nonexercising muscles are required. This consideration of systemic compared with local muscle variation in adiponectin in response to exercise reflects recently published work (Garekani et al. 2011), as does the discovered ability of skeletal muscles to communicate with each other through local myokine releases (Pedersen 2013). Given that background, the aim of this study was to compare the effects of two isocaloric training programs on the adiponectin profiles in exercising and nonexercising muscles each of which have different metabolic features. In addition to studies of quadriceps muscle, we now examine and compare profiles with gastrocnemius muscle, which is defined as an exercising‐glycolytic muscle (Augusto et al. 2004), and with the *masseter* (masticatory) muscle as an example of a muscle which is unlikely to be affected by the exercise regimen and which has been defined as a nonexercising‐glycolytic muscle (Tuxen and Kirkeby 1990; Baverstock et al. 2013).

## Material and Methods

### Ethical approval

The study was approved by the University of Sydney Animal Ethics Committee (Protocol #2015/816). Moreover, this study shares the same ethics approval as a previously published study (Martinez‐Huenchullan et al. 2018) and were carried out according to the guidelines and conform to the principles and regulations, as described in the Editorial (Grundy 2015).

### Animal characteristics

Seventy‐two C57BL/6 10‐week‐old male mice were used in this study (Animal Resources Centre, Perth, Australia). After 1 week of acclimatization mice were randomly divided into two dietary groups (*n* = 36/group) and fed either a high‐fat diet (45% fat) (prepared in‐house as per (Lo et al. 2011)), or standard laboratory chow (12% fat) (Meat free mouse diet, as Specialty feeds ^®^, WA, Australia), ad libitum, for 10 weeks. Simultaneously, each dietary group was then randomized to either constant moderate‐intensity endurance (END) or high‐intensity interval training (HIIT), as previously described (Martinez‐Huenchullan et al. 2018). Untrained animals fed either the Chow or HFD acted as controls. Consequently, six groups (*n* = 12/group) were analyzed during this study: CHOW untrained, CHOW + END, CHOW + HIIT, HFD untrained, HFD + END, and HFD + HIIT. Phenotyping studies including body weight (g) and insulin sensitivity using an insulin tolerance test (ITT) as previously described (Lo et al. 2011), were performed before commencement of diet and exercise and after these interventions just prior to euthanasia. Animals were euthanized by exsanguination through cardiac puncture after administration of isoflurane (3%) in oxygen to induce a deep anesthetic state. *Quadriceps*,* gastrocnemius*, and *masseter* muscles were dissected, collected and appropriately stored at −80°C for later analysis. The *quadriceps* data presented here, as previously stated, has been published in a previous issue of *Physiological Reports* (Martinez‐Huenchullan et al. 2018), and it is included here for the sole purpose of facilitating the comparisons between the three muscles.

### Exercise training

After 1 week of treadmill acclimation (6 m/sec for 10 min), a maximal running capacity (MRC) test was performed. This progressive test started from a speed of 6 m/min and was increased every three min by 3 m/min until exhaustion (Cunha et al. 2012), defined by the inability of the animal to reach the end of the lane after being encouraged with 5 mechanical stimuli (soft brush) delivered within one minute. The final speed was defined as 100% of the MRC and the distance covered was the aerobic performance of the animal. The exercise intensity of the two different training programs was calculated using the initial MRC. The exercise programs were designed to be isocaloric, which was achieved as follows: for END a running session at 70% of the MRC for 40 min, and for HIIT eight bouts (2.5 min each) at 90% of the MRC intercalated by eight active rest periods (2.5 min each) at 50% of the MRC (40 min total per session). Each training program was performed in the morning, three times per week for 10 weeks. Untrained animals were not exposed to any additional exercise.

### RNA extraction, reverse transcription, and real time‐qPCR

Real time‐qPCR to determine tissue mRNA levels was performed using standard methods (Tam et al. 2015). In brief, total RNA was extracted from homogenized muscle tissue weighing 50–60 mg with TRI reagent (Sigma^®^, Cat No. T9424). RNA quantity and quality were measured using a spectrophotometer (Thermo Fisher Scientific, Nanodrop ^®^, Walthan, MA), where a A260/280 ratio above 1.8 was obtained in all samples in order to assure a good RNA quality. The reverse transcription step was performed in each case on 1000 ng of RNA, plus 0.5 *μ*L of oligo(dT) (Life Technologies, Cat No. 18418‐012) at 100 *μ*mol/L, and 0.5 *μ*L of random hexamers (Applied Biosystems, Life Technologies, Cat No. N8080127) at 2 *μ*mol/L. Real time‐qPCR was performed on 5 *μ*L of cDNA with added 7.5 *μ*L of SensiMix SYBR Green (Bioline, Cat No. QT605‐20), 0.5 *μ*L of each primer (forward and reverse) at 10 *μ*mol/L, and 1.5 *μ*L of ddH_2_O. Amplification was achieved using the Rotor‐gene Q thermocycler (QIAGEN ^®^, Hilden, Germany). In each run, a no‐template control was included as a negative control, and a melt curve analysis was performed to confirm the specificity of the reaction. Results are expressed using the delta‐delta Ct corrected for the housekeeper gene Rpl7L1 (Thomas et al. 2014). The genes analyzed along with their primer sequences are as follows: Adiponectin: forward: 5′‐CGACACCAAAAGGGCTCAGG‐3′, reverse: 5′‐ACGTCATCTTCGGCATGACT‐3′; AdipoR1: forward: 5′‐GCAGACAAGAGCAGGAGTGT‐3′, reverse: 5′‐TTGACAAAGCCCTCAGCGAT‐3′; Sirtuin1: forward: 5′‐AGCGGCTTGAGGGTAATCAA‐3′, reverse: 5′‐GAGTATACCTCAGCACCGTGG‐3′; Peroxisome proliferator‐activated receptor gamma coactivator 1‐alpha (Pgc‐1 alpha): forward: 5′‐CTGCGGGATGATGGAGACAG‐3′, reverse: 5′‐TCGTTCGACCTGCGTAAAGT‐3′; Uncoupling protein 2 (Ucp2): forward: 5′‐GGCCTCTGGAAAGGGACTTCT‐3′, reverse: 5′‐TTGGCTTTCAGGAGAGTATCTTT‐3′; Rpl7L1: forward: 5′‐ACGGTGGAGCCTTATGTGAC‐3′, reverse: 5′‐TCCGTCAGAGGGACTGTCTT‐3′. The PCR efficiencies across genes were 1.01 on average, whereas the range of efficiencies was 0.91–1.12.

### Protein analysis by immunoblotting (Western blot)

Protein expression of adiponectin and AdipoR1 were determined in each muscle type by Western immunoblot, using established methods (Tam et al. 2015). In brief, 25–30 mg of muscle was homogenized in ice‐cold buffer containing 50 nmol/L Tris HCl, 150 nmol/L NaCl, 1% Triton X‐100, 0.5% Na‐deoxycholate, and 0.1% SDS. Then, 40 *μ*g of protein was loaded to a precast polyacrylamide gradient gel (4–15%) (Bio‐Rad^®^, Cat No. 4568086). After the proteins were transferred to a nitrocellulose membrane (Bio‐Rad^®^, Cat No. 1704158), these were blocked for 1 h using 5% skim milk in buffer (TBST containing 0.6% Tris HCl w/v, 0.1% Tris‐base w/v, 0.6% NaCl w/v, and 0.05% Tween‐20 v/v). Membranes were incubated in primary antibodies as follows and as per the manufacturer instructions: Adiponectin 1:2000 (GeneTex, Cat No. GTX23455), Adiponectin receptor 1 (AdipoR1) 1:2000 (GeneTex, Cat No. GTX32425) overnight at 4°C. To enable detection membranes were incubated with peroxidase labeled Anti‐rabbit IgG (1:10000, Sigma^®^, Cat No. S9169) for 1 h at room temperature. Finally, the membranes were developed using Clarity™ Western ECL substrate (Bio‐Rad^®^, Cat No. 170‐5061) and visualized using a Chemidoc imaging system (Bio‐Rad^®^, Hercules, CA). Densitometric analysis of the bands was performed using Image Lab software (Bio‐Rad^®^). Protein loading was confirmed and normalized using Ponceau S staining of the whole membrane in each respective experiment. This normalization was performed on raw densitometric data, whereas the normalized values were considered for statistical analysis, considering the CHOW untrained group as control.

### Statistical analysis

Statistical analyses were performed using GraphPad software Version 7.0. Normally distributed data was expressed as mean ± SD. Non‐normally distributed data was expressed as median [interquartile range]. Effects of dietary interventions (CHOW vs. HFD) and the differences between exercise conditions (untrained vs. END vs. HIIT) were calculated using a Two‐way ANOVA analysis with Dunnet's and Sidak's post hoc tests. *P* values < 0.05 were considered statistically significant.

## Results

### Animal phenotyping

The animal phenotypes were published previously (Martinez‐Huenchullan et al. 2018). Briefly, the animals on the HFD were significantly heavier than chow fed mice (*P* < 0.05) and both exercise interventions similarly prevented the weight gain (both *P* < 0.05). Hyperglycemia and hyperinsulinemia driven by HFD were only prevented by END (both *P* < 0.05). The insulin tolerance test, showed a similar blood excursion for glucose, indicating a more insulin responsive state (both *P* < 0.05), after END and HIIT.

### Adiponectin induction and adiponectin receptor 1 in quadriceps, gastrocnemius, and masseter

**Figure 1:**
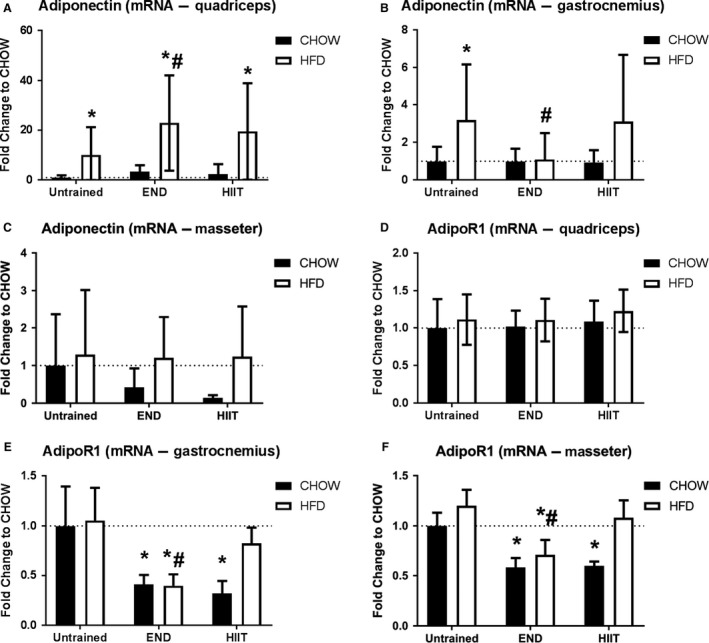
Muscle adiponectin and adiponectin receptor 1 (AdipoR1) mRNA levels from three different muscles. The mRNA levels of adiponectin from *quadriceps* (A), *gastrocmenius* (B), and *masseter* (C), and also, muscle AdipoR1 mRNA levels from *quadriceps* (D), *gastrocnemius* (E), and *masseter* (F) muscles, are presented. Data are shown as mean ± SD; the number of animals per group is 6‐11. * *P* < 0.05 versus CHOW untrained; #: *P* < 0.05 versus HFD untrained by Two‐way ANOVA with Dunnet's and Sidak's post hoc tests.

In *quadriceps* HFD and exercise, particularly END, induced an increase in adiponectin mRNA (Fig. 1A; *P* < 0.05). Interestingly, in *gastrocnemius* this HFD‐driven increase was prevented by END but not by HIIT (Fig. 1B). No effect of HFD or exercise was seen in adiponectin mRNA in the nonexercising *masseter* muscles (Fig. 1C; *P* > 0.05). The pattern for AdipoR1 mRNA was different, in HFD mice where END but not HIIT produced a decrease in both *gastrocnemius* and *masseter* AdipoR1 mRNA level (Fig. 1E–F; *P* < 0.05) with no changes in *quadriceps* (Fig. 1D; *P* > 0.05).

**Figure 2:**
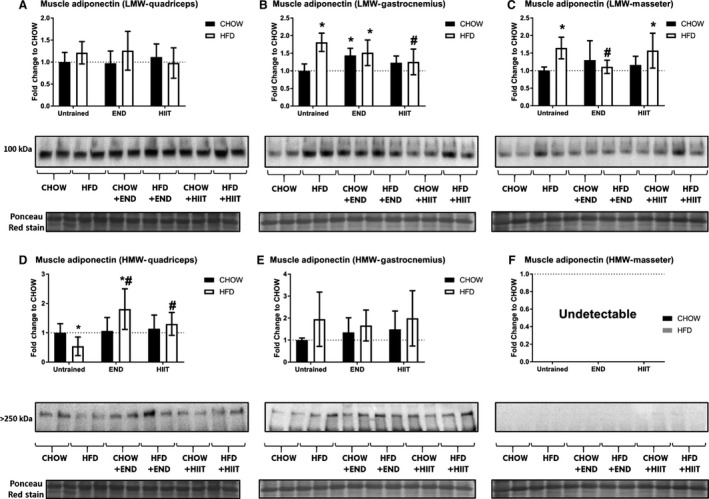
Protein levels of muscle adiponectin isoform from three different muscles. Muscle low‐molecular weight (LMW) adiponectin from quadriceps (A), gastrocnemius (B), and masseter (C), and high‐molecular weight (HMW) adiponectin from the same muscles (D–F) are presented. Also, their representative blots are exhibited below the respective graph with the membrane stained with Ponceau S used as loading control. Data are presented as mean ± SD; the number of animals in each group is 6–8. *P < 0.05 versus CHOW untrained; #: *P* < 0.05 versus HFD untrained by Two‐way ANOVA with Dunnet's and Sidak's post hoc tests. *Quadriceps* data previously published (Martinez‐Huenchullan et al. 2018).

From a protein perspective, low‐molecular weight (LMW) muscle adiponectin was increased by HFD in *gastrocnemius* and *masseter* muscles (*P* < 0.05), without changes in *quadriceps* (Fig. 2A). Interestingly, END increased this isoform in the *gastrocnemius* of Chow fed animals (*P* < 0.05), but not in HFD animals (Fig. 2B). In *masseter* muscles, the HFD‐driven increase in LMW muscle adiponectin was prevented by END (Fig. 2C; *P* < 0.05). As previously reported, the major changes in the HMW isoform of muscle adiponectin were seen in *quadriceps* (Fig. 2D; *P* < 0.05), but not in *gastrocnemius* (Fig. 2E; *P* > 0.05). In *masseter* muscle this isoform was not detectable by our methods (Fig. 2F).

**Figure 3:**
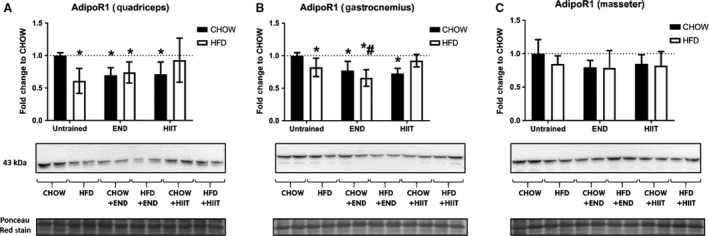
Protein levels of muscle adiponectin receptor 1 (AdipoR1) from three different muscles. Muscle AdipoR1 from quadriceps (A), gastrocnemius (B), and masseter (C), are presented. Their representative Western immunoblots are exhibited below the respective graph with each membrane stained with Ponceau S used as loading control. Data are presented as mean ± SD and the number of animals in each group is 6–8. * *P* < 0.05 versus CHOW untrained; #: *P* < 0.05 versus HFD untrained by Two‐way ANOVA with Dunnet's and Sidak's post hoc tests.

Protein levels of muscle AdipoR1 were significantly decreased by diet and/or exercise in *quadriceps* and *gastrocnemius* (*P* < 0.05). Interestingly, this change was only seen in HFD animals that underwent END training (Fig. 3A–B; *P* < 0.05). In *masseter* muscles, no major diet nor exercise effects were detected (Fig. 3C; *P* > 0.05).

### Adiponectin downstream factors

**Figure 4:**
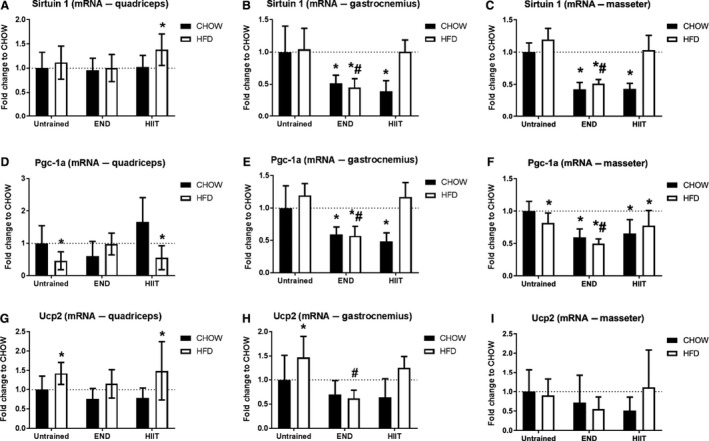
Muscle adiponectin downstream factors mRNA levels from three different muscles. Quadriceps, gastrocnemius, and masseter mRNA levels of sirtuin 1 (A–C), peroxisome proliferator‐activated receptor gamma coactivator 1‐alpha (Pgc‐1 alpha; D–F), and uncoupling protein 2 (Ucp‐1; G–I) are shown. Data are presented as mean ± SD and the number of animals per group is 6‐11. *P < 0.05 versus CHOW untrained; #: *P* < 0.05 versus HFD untrained by Two‐way ANOVA with Dunnet's and Sidak's post hoc tests.

In *gastrocnemius* and *masseter* muscles, both exercise programs induced a decrease in the mRNA level of sirtuin 1 (*P* < 0.05), changes that were not seen in HFD animals under HIIT. There were no diet or exercise induced effects on *quadriceps* muscle (Fig. 4A–C; *P* > 0.05). HFD induced a decrease in Pgc‐1a mRNA levels in *quadriceps* and *masseter* muscles (*P* < 0.05), whereas both training programs decreased Pgc‐1a mRNA levels in Chow fed animals in *gastrocnemius* and *masseter* muscles (Fig. 4D–F; *P* < 0.05). HFD induced a significant increase in Ucp2 mRNA levels in exercising muscles (*quadriceps* and *gastrocnemius*;* P* < 0.05), which was prevented by END but not HIIT (Fig. 4G–I).

## Discussion

The intent of this study was to compare the effects of diet (HFD and chow), and of two isocaloric exercise programs on the skeletal muscle adiponectin profiles of *quadriceps, gastrocnemius, and masseter* muscles. We describe that the most bioactive adiponectin isoform (the HMW form), was highly affected by exercise in muscles with a higher proportion of oxidative fibers (*quadriceps*). Moreover, the less bioactive isoform (the LMW form), is more influenced by exercise in glycolytic muscles (e.g., *gastrocnemius*). In contrast, change in nonexercising muscles (e.g., *masseter*) seems to be influenced by diet but not exercise. Moreover, in the three muscles analyzed, END was superior in improving this response compared to HIIT in a HFD context. Interestingly, no expression of HMW muscle adiponectin was found in nonexercising muscles (*masseter*), suggesting a major influence of exercise in the expression of this isoform.

Over the last two decades, adiponectin production has been described in muscle cells in vitro and in vivo (Delaigle et al. 2004; Krause et al. 2008; Liu et al. 2009; Goto et al. 2013). Interestingly, this phenomenon seems to fulfill an autocrine/paracrine role (Garekani et al. 2011), particularly in response to metabolic stressors, such as inflammatory cytokines (Delaigle et al. 2004), caloric restriction/exercise (Dai et al. 2013), and HFD (Jortay et al. 2012). Given its anti‐inflammatory, anti‐oxidative, and anti‐apoptotic features (Jortay et al. 2010, 2012) increased muscle adiponectin have been explained mechanistically as a protective response in tissues against metabolic insults. This concept was primarily investigated in adiponectin KO mice, where an intraperitoneal injection of LPS increased the levels of inflammatory and oxidative damage in their *tibialis anterior* muscles. Interestingly, these mice were rescued by local adiponectin administration (Jortay et al. 2010). In our study, we found that HFD decreased muscle adiponectin in *quadriceps* (mixed‐fiber type muscle), in contrast with the mainly glycolytic *gastrocnemius* and *masseter* muscles (Tuxen and Kirkeby 1990; Augusto et al. 2004). These muscles exhibited the opposite response in HFD untrained animals, where increases in lower molecular mass adiponectin isoforms (LMW) were seen. These findings are concordant with the proposal that adiponectin induction seems not to be homogeneous across muscles. Krause et al. described that muscle adiponectin was particularly present in fibers rich in intramyocellular lipids, which were associated with oxidative metabolism. It has been suggested that muscle adiponectin can influence muscle phenotype of wild‐type mice against adiponectin‐KO animals as their *tibialis anterior* muscles had higher type IIB (mostly glycolytic) fiber area (Krause et al. 2008). These factors may be the reason why in the literature conflicting results can be found in published reports. For example, three different studies found increased muscle adiponectin after metabolic insults (high fat/sucrose diet or genetic models of obesity (*ob/ob* mice)), and based their conclusions on the analysis of glycolytic muscles such as *tibialis anterior* and *gastrocnemius* (Delaigle et al. 2006; Bonnard et al. 2008; Jortay et al. 2012). Others have reported decreases in HMW muscle adiponectin in *db/db* mouse *soleus*, a highly oxidative muscle (Liu et al. 2009). The mechanisms behind the discrepancy are unknown, however, the length of dietary interventions seems to be also relevant, given that after 20 weeks of high‐fat/sucrose diet, the *gastrocnemius* of rats also showed lower levels of muscle adiponectin (Yang et al. 2006).

In all three muscles analyzed in this current work, HFD‐driven changes in adiponectin profiles were more effectively prevented by END compared with HIIT. Furthermore, END increased LMW muscle adiponectin in *gastrocnemius* of lean animals, suggesting that this preferential effect of exercise was independent of diet (Chow or HFD). The evidence linking the effects of exercise on muscle adiponectin was scarce, however, increases of adiponectin in *gastrocnemius* were seen after 6 months of exercise training in lean rats (Dai et al. 2013), effects that were comparable to caloric restriction for a similar amount of time. Their exercise protocol was similar to our END program, but with more sessions per week (five). This supported the suggestion that END, compared with HIIT, can effectively increase muscle adiponectin production. An effect that might, at least partially, explain the better metabolic effects of this type of training in a prevention context (Martinez‐Huenchullan et al. 2018). Nevertheless, further mechanistic studies in this topic are urgently needed, in order to elucidate the pathways responsible for the differential effect of the two training programs.

It is well understood that adiponectin exerts its action through adiponectin receptors, and two isoforms have been identified (AdipoR1‐2) (Liu and Sweeney 2014). AdipoR1 is the most abundant receptor in skeletal muscle and it has been identified as a mediator of the metabolic effects of adiponectin in this tissue (Jortay et al. 2012), whereas AdipoR2 does not seem to have a major role in skeletal muscle (Goto et al. 2013). In the context of obesity, adiponectin resistance has been suggested as another mechanism behind the metabolic dysfunction found in skeletal muscle (Liu and Sweeney 2014), characterized by a decrease in muscle AdipoR1 (Fang et al. 2005; Bonnard et al. 2008) and impairing adiponectin signaling. In our study, similar decreases of AdipoR1 were found in *quadriceps* and *gastrocnemius* of HFD‐untrained animals. Interestingly, both types of exercise did not prevent this change, where a decrease of this protein was also seen in trained lean animals, suggesting that this finding is a physiological adaptation to exercise. In this context, other studies have described opposite effects of exercise, where increases of this protein have been described (Dai et al. 2013; Pierard et al. 2016). However, in those studies, samples were collected 24 h after the last training session, which contrast with our study where we euthanized the animals 72 h after their last training session, in order to prevent “carry over” effects from recent acute exercise. Nevertheless, this point requires further investigation.

To further address if there are changes in muscle adiponectin downstream signaling markers in our study, we explored the mRNA levels of three known downstream factors of adiponectin, namely sirtuin 1, Pgc‐1a, and Ucp2 (Iwabu et al. 2010; Liu and Sweeney 2014). HFD decreased the levels of Pgc‐1a (*quadriceps*) and increased the expression of Ucp2 (*quadriceps* and *gastrocnemius*), which aligns with what has been described in the literature (Schrauwen et al. 2001; Sparks et al. 2005), suggesting that HFD was effectively impairing adiponectin signaling in our model. We support this statement by considering that decreases in PGC‐1ɑ protein have been found in muscles where adiponectin signaling has been muscle‐specifically disrupted by knocking out the AdipoR1 gene (Iwabu et al. 2010). Therefore, we propose that the HFD‐driven decreases in HMW adiponectin in combination with decreases of AdipoR1 protein are mechanisms behind the lower levels of Pgc‐1a mRNA levels found in *quadriceps* of HFD untrained animals. Interestingly, only END training was able to prevent those changes, which might mean that aerobic exercise programs are more effective regimens in the context of preventing HFD derived‐metabolic dysfunction in skeletal muscle, in contrast to anaerobic/aerobic programs such as HIIT. Nevertheless, in the case of sirtuin 1 and Pgc‐1a we identified that exercise reduced the mRNA of these genes in *gastrocnemius* and *masseter*, a finding that will require further exploration in future studies.

In conclusion, our results indicate that diet, muscle type/activity, and exercise type each influence muscle adiponectin profile, its receptor 1, and some of its downstream factors. Therefore, these parameters should be taken into consideration at the time of investigating this endocrine response of skeletal muscle, particularly in the context of obesity and metabolic disorders.

## Conflict of Interest

The authors declare that there is no conflict of interest.

## References

[phy213848-bib-0001] Augusto, V. , C. Podovani , and G. Rocha‐Campos . 2004 Skeletal muscle fiber types in C57BL6J mice. Braz. J. Morphol. Sci. 21:89–94.

[phy213848-bib-0002] Baverstock, H. , N. S. Jeffery , and S. N. Cobb . 2013 The morphology of the mouse masticatory musculature. J. Anat. 223:46–60. 23692055 10.1111/joa.12059 PMC4487762

[phy213848-bib-0003] Bonnard, C. , A. Durand , H. Vidal , and J. Rieusset . 2008 Changes in adiponectin, its receptors and AMPK activity in tissues of diet‐induced diabetic mice. Diabetes Metab. 34:52–61. 18222103 10.1016/j.diabet.2007.09.006

[phy213848-bib-0004] Cunha, T. F. , J. B. Moreira , N. A. Paixao , J. C. Campos , A. W. Monteiro , A. V. Bacurau , et al. 2012 Aerobic exercise training upregulates skeletal muscle calpain and ubiquitin‐proteasome systems in healthy mice. J. Appl. Physiol. 1985:1839–1846. 10.1152/japplphysiol.00346.2011 22461440

[phy213848-bib-0005] Dai, Y. , J. Pang , H. Gong , W. Fan , and T. M. Zhang . 2013 Roles and tissue source of adiponectin involved in lifestyle modifications. J. Gerontol. A Biol. Sci. Med. Sci. 68:117–128. 22562959 10.1093/gerona/gls131

[phy213848-bib-0006] Delaigle, A. M. , J. C. Jonas , I. B. Bauche , O. Cornu , and S. M. Brichard . 2004 Induction of adiponectin in skeletal muscle by inflammatory cytokines: in vivo and in vitro studies. Endocrinology 145:5589–5597. 15319349 10.1210/en.2004-0503

[phy213848-bib-0007] Delaigle, A. M. , M. Senou , Y. Guiot , M. C. Many , and S. M. Brichard . 2006 Induction of adiponectin in skeletal muscle of type 2 diabetic mice: in vivo and in vitro studies. Diabetologia 49:1311–1323. 16570160 10.1007/s00125-006-0210-y

[phy213848-bib-0008] Fang, X. , R. Palanivel , X. Zhou , Y. Liu , A. Xu , Y. Wang , et al. 2005 Hyperglycemia‐ and hyperinsulinemia‐induced alteration of adiponectin receptor expression and adiponectin effects in L6 myoblasts. J. Mol. Endocrinol. 35:465–476. 16326833 10.1677/jme.1.01877

[phy213848-bib-0009] Garekani, E. T. , H. Mohebbi , R. R. Kraemer , and R. Fathi . 2011 Exercise training intensity/volume affects plasma and tissue adiponectin concentrations in the male rat. Peptides 32:1008–1012. 21291933 10.1016/j.peptides.2011.01.027

[phy213848-bib-0010] Goto, A. , Y. Ohno , A. Ikuta , M. Suzuki , T. Ohira , T. Egawa , et al. 2013 Up‐regulation of adiponectin expression in antigravitational soleus muscle in response to unloading followed by reloading, and functional overloading in mice. PLoS ONE 8:e81929. 24324732 10.1371/journal.pone.0081929 PMC3855747

[phy213848-bib-0011] Grundy, D. 2015 Principles and standards for reporting animal experiments in The Journal of Physiology and Experimental Physiology. Exp. Physiol. 100:755–758. 26076765 10.1113/EP085299

[phy213848-bib-0012] Iwabu, M. , T. Yamauchi , M. Okada‐Iwabu , K. Sato , T. Nakagawa , M. Funata , et al. 2010 Adiponectin and AdipoR1 regulate PGC‐1alpha and mitochondria by Ca(2 + ) and AMPK/SIRT1. Nature 464:1313–1319. 20357764 10.1038/nature08991

[phy213848-bib-0013] Jacobs, R. A. , V. Diaz , A. K. Meinild , M. Gassmann , and C. Lundby . 2013 The C57Bl/6 mouse serves as a suitable model of human skeletal muscle mitochondrial function. Exp. Physiol. 98:908–921. 23180810 10.1113/expphysiol.2012.070037

[phy213848-bib-0014] Jortay, J. , M. Senou , A. Delaigle , L. Noel , T. Funahashi , N. Maeda , et al. 2010 Local induction of adiponectin reduces lipopolysaccharide‐triggered skeletal muscle damage. Endocrinology 151:4840–4851. 20702578 10.1210/en.2009-1462

[phy213848-bib-0015] Jortay, J. , M. Senou , M. Abou‐Samra , L. Noel , A. Robert , M. C. Many , et al. 2012 Adiponectin and skeletal muscle: pathophysiological implications in metabolic stress. Am. J. Pathol. 181:245–256. 22658482 10.1016/j.ajpath.2012.03.035

[phy213848-bib-0016] Krause, M. P. , Y. Liu , V. Vu , L. Chan , A. Xu , M. C. Riddell , et al. 2008 Adiponectin is expressed by skeletal muscle fibers and influences muscle phenotype and function. Am. J. Physiol. Cell Physiol. 295:C203–C212. 18463233 10.1152/ajpcell.00030.2008 PMC2493546

[phy213848-bib-0017] Liu, Y. , and G. Sweeney . 2014 Adiponectin action in skeletal muscle. Best Pract. Res. Clin. Endocrinol. Metab. 28:33–41. 24417944 10.1016/j.beem.2013.08.003

[phy213848-bib-0018] Liu, Y. , S. Chewchuk , C. Lavigne , S. Brule , G. Pilon , V. Houde , et al. 2009 Functional significance of skeletal muscle adiponectin production, changes in animal models of obesity and diabetes, and regulation by rosiglitazone treatment. Am. J. Physiol. Endocrinol. Metab. 297:E657–E664. 19531641 10.1152/ajpendo.00186.2009

[phy213848-bib-0019] Lo, L. , S. V. McLennan , P. F. Williams , J. Bonner , S. Chowdhury , G. W. McCaughan , et al. 2011 Diabetes is a progression factor for hepatic fibrosis in a high fat fed mouse obesity model of non‐alcoholic steatohepatitis. J. Hepatol. 55:435–444. 21184785 10.1016/j.jhep.2010.10.039

[phy213848-bib-0020] Martinez‐Huenchullan, S. F. , B. R. Maharjan , P. F. Williams , C. S. Tam , S. V. McLennan , and S. M. Twigg . 2018 Differential metabolic effects of constant moderate versus high intensity interval training in high‐fat fed mice: possible role of muscle adiponectin. Physiol. Rep. 6: https://doi.org/10.14814/phy2.13599. 10.14814/phy2.13599 PMC5812883 29446245

[phy213848-bib-0021] Pedersen, B. K. 2013 Muscle as a secretory organ. Compr. Physiol. 3:1337–1362. 23897689 10.1002/cphy.c120033

[phy213848-bib-0022] Pierard, M. , S. Conotte , A. Tassin , S. Boutry , P. Uzureau , K. Z. Boudjeltia , et al. 2016 Interactions of exercise training and high‐fat diet on adiponectin forms and muscle receptors in mice. Nutr. Metab. (Lond) 13:75. 27822289 10.1186/s12986-016-0138-2 PMC5094086

[phy213848-bib-0023] Schrauwen, P. , H. Hoppeler , R. Billeter , A. H. Bakker , and D. R. Pendergast . 2001 Fiber type dependent upregulation of human skeletal muscle UCP2 and UCP3 mRNA expression by high‐fat diet. Int. J. Obes. Relat. Metab. Disord. 25:449–456. 11319645 10.1038/sj.ijo.0801566

[phy213848-bib-0024] Sparks, L. M. , H. Xie , R. A. Koza , R. Mynatt , M. W. Hulver , G. A. Bray , et al. 2005 A high‐fat diet coordinately downregulates genes required for mitochondrial oxidative phosphorylation in skeletal muscle. Diabetes 54:1926–1933. 15983191 10.2337/diabetes.54.7.1926

[phy213848-bib-0025] Tam, C. S. , J. E. Power , T. P. Markovic , C. Yee , M. Morsch , S. V. McLennan , et al. 2015 The effects of high‐fat feeding on physical function and skeletal muscle extracellular matrix. Nutr. Diabetes 5:e187. 26657013 10.1038/nutd.2015.39 PMC4735053

[phy213848-bib-0026] Thomas, K. C. , X. F. Zheng , F. Garces Suarez , J. M. Raftery , K. G. Quinlan , N. Yang , et al. 2014 Evidence based selection of commonly used RT‐qPCR reference genes for the analysis of mouse skeletal muscle. PLoS ONE 9:e88653. 24523926 10.1371/journal.pone.0088653 PMC3921188

[phy213848-bib-0027] Tomlinson, D. J. , R. M. Erskine , K. Winwood , C. I. Morse , and G. L. Onambele . 2014 Obesity decreases both whole muscle and fascicle strength in young females but only exacerbates the aging‐related whole muscle level asthenia. Physiol. Rep. 2: https://doi.org/10.14814/phy2.12030. 10.14814/phy2.12030 PMC4208641 24963030

[phy213848-bib-0028] Tuxen, A. , and S. Kirkeby . 1990 An animal model for human masseter muscle: histochemical characterization of mouse, rat, rabbit, cat, dog, pig, and cow masseter muscle. J. Oral Maxillofac. Surg. 48:1063–1067. 1698954 10.1016/0278-2391(90)90290-i

[phy213848-bib-0029] Yang, B. , L. Chen , Y. Qian , J. A. Triantafillou , J. A. McNulty , K. Carrick , et al. 2006 Changes of skeletal muscle adiponectin content in diet‐induced insulin resistant rats. Biochem. Biophys. Res. Commun. 341:209–217. 16414018 10.1016/j.bbrc.2005.12.172

